# Reactivation of latent HIV-1 provirus via targeting protein phosphatase-1

**DOI:** 10.1186/s12977-015-0190-4

**Published:** 2015-07-16

**Authors:** Mudit Tyagi, Sergey Iordanskiy, Tatyana Ammosova, Namita Kumari, Kahli Smith, Denitra Breuer, Andrey V Ilatovskiy, Yasemin Saygideğer Kont, Andrey Ivanov, Aykut Üren, Dmytro Kovalskyy, Michael Petukhov, Fatah Kashanchi, Sergei Nekhai

**Affiliations:** Department of Medicine, The George Washington University, Washington, DC, 2003 USA; National Center for Biodefense and Infectious Diseases, George Mason University, Manassas, VA 20110 USA; Center for Sickle Cell Disease, Howard University, 1840 7th Street, N.W. HURB1, Suite 202, Washington, DC, 20059 USA; Department of Medicine, Howard University, Washington, DC, 20059 USA; Yakut Science Center for Complex Medical Problems, Yakutsk, 677019 Russia; Division of Molecular and Radiation Biophysics, Petersburg Nuclear Physics Institute, Gatchina, Russia; Instiute of Nanobiotechnologies, St. Petersburg State Polytechnical University, St. Petersburg, Russia; Lombardi Comprehensive Cancer Center, Georgetown University, Washington, DC, 20057 USA; Department of Biochemistry and Cancer Therapy and Research Center, University of Texas Health Science Center at San Antonio, 7703 Floyd Curl Drive, San Antonio, TX 78229 USA

## Abstract

**Background:**

HIV-1 escapes antiretroviral drugs by integrating into the host DNA and forming a latent transcriptionally silent HIV-1 provirus. This provirus presents the major hurdle in HIV-1 eradication and cure. Transcriptional activation, which is prerequisite for reactivation and the eradication of latent proviruses, is impaired in latently infected T cells due to the lack of host transcription factors, primarily NF-κB and P-TEFb (CDK9/cyclin T1). We and others previously showed that protein phosphatase-1 (PP1) regulates HIV-1 transcription by modulating CDK9 phosphorylation. Recently we have developed a panel of small molecular compounds targeting a non-catalytic site of PP1.

**Results:**

Here we generated a new class of sulfonamide-containing compounds that activated HIV-1 in acute and latently infected cells. Among the tested molecules, a small molecule activator of PP1 (SMAPP1) induced both HIV-1 replication and reactivation of latent HIV-1 in chronically infected cultured and primary cells. In vitro, SMAPP1 interacted with PP1 and increased PP1 activity toward a recombinant substrate. Treatment with SMAPP1 increased phosphorylation of CDK9’s Ser90 and Thr186 residues, but not Ser175. Proteomic analysis showed upregulation of P-TEFb and PP1 related proteins, including PP1 regulatory subunit Sds22 in SMAPP1-treated T cells. Docking analysis identified a PP1 binding site for SMAPP1 located within the C-terminal binding pocket of PP1.

**Conclusion:**

We identified a novel class of PP1-targeting compounds that reactivate latent HIV-1 provirus by targeting PP1, increasing CDK9 phosphorylation and enhancing HIV transcription. This compound represents a novel candidate for anti-HIV-1 therapeutics aiming at eradication of latent HIV-1 reservoirs.

## Background

Despite efficient antiretroviral therapy, eradication of human immunodeficiency virus (HIV) 1 infection is challenging and requires novel biological insights and therapeutic strategies. Eradication of latent HIV-1 provirus is especially challenging as integrated HIV-1 is not affected by the existing anti-HIV-1 drugs unless viral transcription is activated [1]. Efficient HIV-1 transcription from HIV-1 long terminal repeat (LTR) requires both host cell factors and HIV-1 Tat protein [2]. HIV-1 Tat protein recruits the positive transcription elongation factor b (P-TEFb), a heterodimeric complex consisting mainly of cell cycle-dependent kinase (CDK) 9 and cyclin T1, to the transactivation response (TAR) RNA [3]. Separately, Tat also recruits histone acetyl transferases (HATs) [4–6] and SWI/SNF remodeling complex [7] to induce transcription from the integrated HIV-1 promoter. P-TEFb activity is repressed by the chicken ovalbumin upstream promoter transcription factor (COUP-TF) interacting protein 2 (STIP2) which also represses HIV-1 promoter and blocks HIV-1 transcription in microglia [8]. STIP2-repressed P-TEFb is recruited to HIV-1 and cellular promoters by high mobility group AT-hook 1 (HMGA1) protein [9]. P-TEFb triggers HIV-1 transcriptional elongation via the phosphorylation of the C-terminal domain (CTD) of RNA polymerase II (RNAPII), the negative elongation factor (NELF) and the DRB-sensitivity inducing complex (DSIF/Spt4/Spt5) [1, 10]. P-TEFb in the cells exists in the form of distinct molecular weight complexes [11]. A low molecular weight, functionally active kinase consists of CDK9 and cyclin T1 subunits [10]. However, the enzymatically inactive, high molecular weight complex carries other additional factors, including 7SK RNA, HEXIM1 protein, La-related LARP7 protein [12–14] and the methylphosphatase capping enzyme MePCE [15, 16]. The high molecular weight complex serves as a source of P-TEFb, from which HIV-1 Tat extracts P-TEFb and recruits it to HIV-1 LTR [17]. Subsequently, Tat facilitates the formation of super-elongation complex (SEC) at HIV-1 LTR, which, in addition to P-TEFb, also carries additional elongation factors and co-activators [18, 19]. Enzymatic activity of P-TEFb and its interaction with Tat is regulated by phosphorylation of CDK serine/threonine residues located in the regulatory T-loop [11]. Phosphorylation of CDK9 at Thr186 is required for its enzymatic activity [20, 21]. We and others have previously shown that protein phosphatase-1 (PP1) dephosphorylates CDK9’s Thr 186 [22, 23]. Moreover, we also showed that PP1 dephosphorylates CDK9’s Ser 175 [22]. A recent study by Jonathan Karn and colleagues showed that phosphorylation of CDK9 Ser175 occurs during the induction of latent HIV-1 provirus and that Tat Lys12 forms a hydrogen bond with CDK9’s phospho-Ser175 [24]. Thus, interaction between Lys12 of Tat and phosphorylated CDK9’s Ser175 facilitates the binding of Tat to P-TEFb [24]. We have recently demonstrated that phosphorylation of CDK9 at Ser90 by CDK2 alters CDK9 association with 7SK snRNP and unregulates HIV-1 transcription [25]. PP1 holoenzyme consists of a constant catalytic subunit (PP1) and a variable PP1 interacting subunit such as NIPP1, PNUTS, Sds22 and others [26]. A Lego-like multicenter interaction of the PP1 catalytic subunit and its various regulatory subunits defines the cellular localization, catalytic activity, and substrate-specificity of the PP1 holoenzyme [27]. Recently, CDK9/cyclin T1 was shown to associate with the PP1 regulatory subunit, PNUTS, and siRNA-mediated knockdown of PNUTS upregulated HIV-1 transcription [28]. Moreover, sequestration of PP1 through the expression of nuclear inhibitor of PP1 reduced HIV-1 transcription [29].

Thus, studies from our group and others showed that PP1 is an important regulator of HIV-1 transcription. We recently developed a panel of small molecular compounds targeted to a non-catalytic site of PP1 and identified 1H4 compound that efficiently inhibited HIV-1 transcription and replication [30]. We further modified 1H4 compound and obtained more potent HIV-1 inhibitors, including 1E7-03 compound [31]. Along with 1,2,3,4-tetrahydracridine series (1H4 derivatives) we evaluated other chemical scaffolds and found that some of these enhanced HIV-1 replication. These compounds contained sulfonamide linker and were structurally distinct from 1,2,3,4-tetrahydracridine series. Here, we characterized the effect of the HIV-1 activators using productively and latently infected cultured and primary cells. The most promising molecule, compound 3, which was renamed as a small molecule activator of PP1 (SMAPP1), induced HIV-1 during single round replication in T cells and in latently infected Jurkat T cells, THP-1 cells and latently infected primary CD4+ T cells. Furthermore, SMAPP1 induced HIV-1 replication in primary PMBCs and latently infected ACH-2 and OM 10.1 cells. In vitro analysis showed that SMAPP1 binds to PP1 in vitro using surface plasmon resonance-based Biacore assay and enhanced dephosphorylation of recombinant PP1 substrate by purified PP1. SMAPP1 specifically enhanced CDK9 phosphorylation at Ser90 and Thr186 residues, but had no effect on CDK9 Ser175 residue phosphorylation as determined with phospho-specific antibodies. Proteomic analysis showed that SMAPP1 increased expression of P-TEFb and PP1-related proteins including a PP1-regulatory subunit, Sds22, which was confirmed by immunoblotting analysis. In silico molecular docking of SMAPP1 showed its preferable interaction with PP1 via the binding site that is located in the C-terminal groove of PP1. Thus, our results indicate that SMAPP1 increased CDK9 phosphorylation and upregulated HIV-1 transcription that led to the reactivation of latent HIV-1 provirus. Eventually all these events translated into enhanced HIV-1 replication and reactivation of latent proviruses in cellular models of HIV latency. Hence, our study identified a novel class of PP1-targeted compounds that activate latent HIV-1 provirus and that may be useful for future anti-HIV-1 therapeutics targeting HIV-1 eradication.

## Results

### Activation of HIV-1 by protein phosphatase-1 (PP1)-targeting compounds

**Figure 1:**
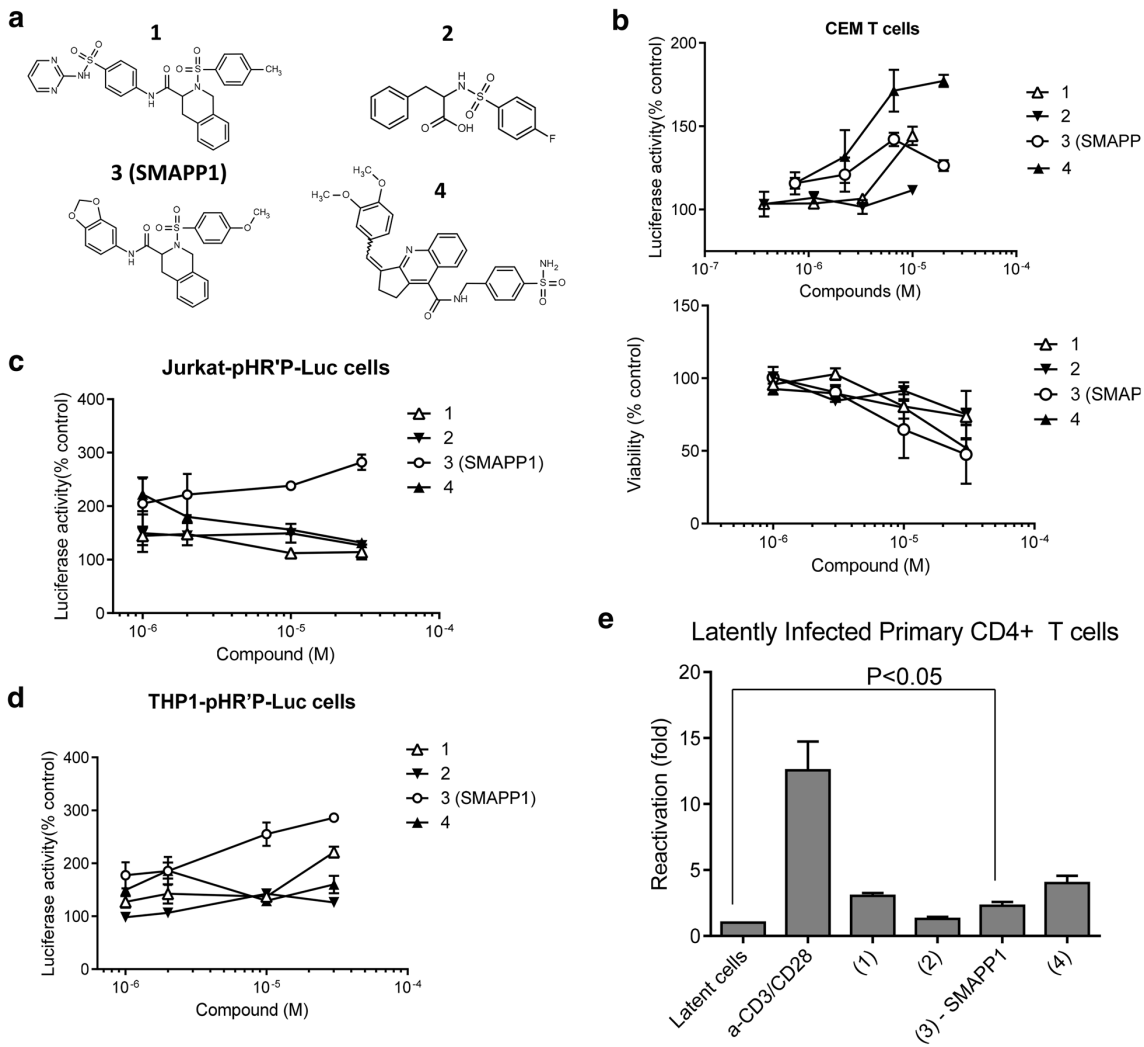
Activation of HIV-1 by PP1-targeting compounds. **a** Chemical structures of compounds 1–4 are shown. **b** Activation of single round HIV-1 replication in CEM T cells. *Upper panel* CEM T cells were infected with VSVG-pseudotyped pNL4-3.Luc.R-E- (HIV-1 Luc) virus for 18 h and then treated for 24 h with the indicated concentrations of the compounds. The cells were lyzed and luciferase activity was measured. *Lower panel* toxicity of compounds was determined with trypan blue exclusion assay using an automated cell counter. **c**, **d** Activation of HIV-1 in latently infected Jurkat T cells and THP-1 cells. Latently infected Jurkat and THP cells were treated with the indicated concentrations of compounds for 48 h. The cells were lyzed and luciferase activity was measured. **e** Activation of HIV-1 in latently infected primary CD4^+^ T cells. The latently infected primary CD4+ T cells, harboring latent HIV provirus (pHR’P-PNL-H13LTat-δNef-GFP) with GFP reporter under LTR promoter, were treated with 5 µM of each drug in triplicate. The GFP expressing cells were assessed and quantified. TCR stimulation with anti-CD3/CD28 antibodies was used as positive control. The results depict the mean of different assays with standard deviation.

We recently developed a small molecule, 1E7-03, that targets a non-catalytic site of PP1, interfered with the binding of Tat to PP1 and inhibited HIV-1 transcription and replication [31]. The 1E7-03 compound is a 4-benzylidene-1,2,3,4-tetrahydracridine with flexible carboxylic tail at position 9. We conducted a pharmacophore search for alternative chemical scaffolds to mimic aromatic moiety of the acridine scaffold and found that sulfonamides may serve as putative analogs. For instance, 2-(benzenesulfonyl)-3,4-dihydro-1H-isoquinoline matched aromatic option of the 1H4 (Figure 1a). Such compounds and their analogs available from Enamine stock were grouped in a library containing 38 new compounds which were screened by utilizing a single round HIV-1 infection assay using CEM T cells infected with VSVG-pseudotyped HIV-1 virus expressing luciferase (HIV-1 Luc) as previously described [32]. Several compounds (Figure 1a, compounds 1, 3 and 4) enhanced HIV-1 infection (Figure 1b, upper panel) without showing significant toxicity (Figure 1b, lower panel). The compounds were further tested in Jurkat and THP-1 cells latently infected with HIV-1 [33, 34]. In both lymphocyte and macrophage cell lines, HIV-1 was activated by compound 3 (Figure 1c, d). We next tested the effect of the compounds on HIV-1 activation in latently infected primary CD4+ T cells (see details in “Methods”). SMAPP1, as well as compounds 1 and 4, activated HIV-1 (Figure 1e), suggesting the utility of these compounds during kick-and-kill cure strategy. Based on these observations, we chose compound 3 (and renamed it as small molecule activating PP1—SMAPP1 for further analysis because it consistently activated HIV-1 in all tested conditions.

### SMAPP1 shows no toxicity and activates HIV-1 gene expression

**Figure 2:**
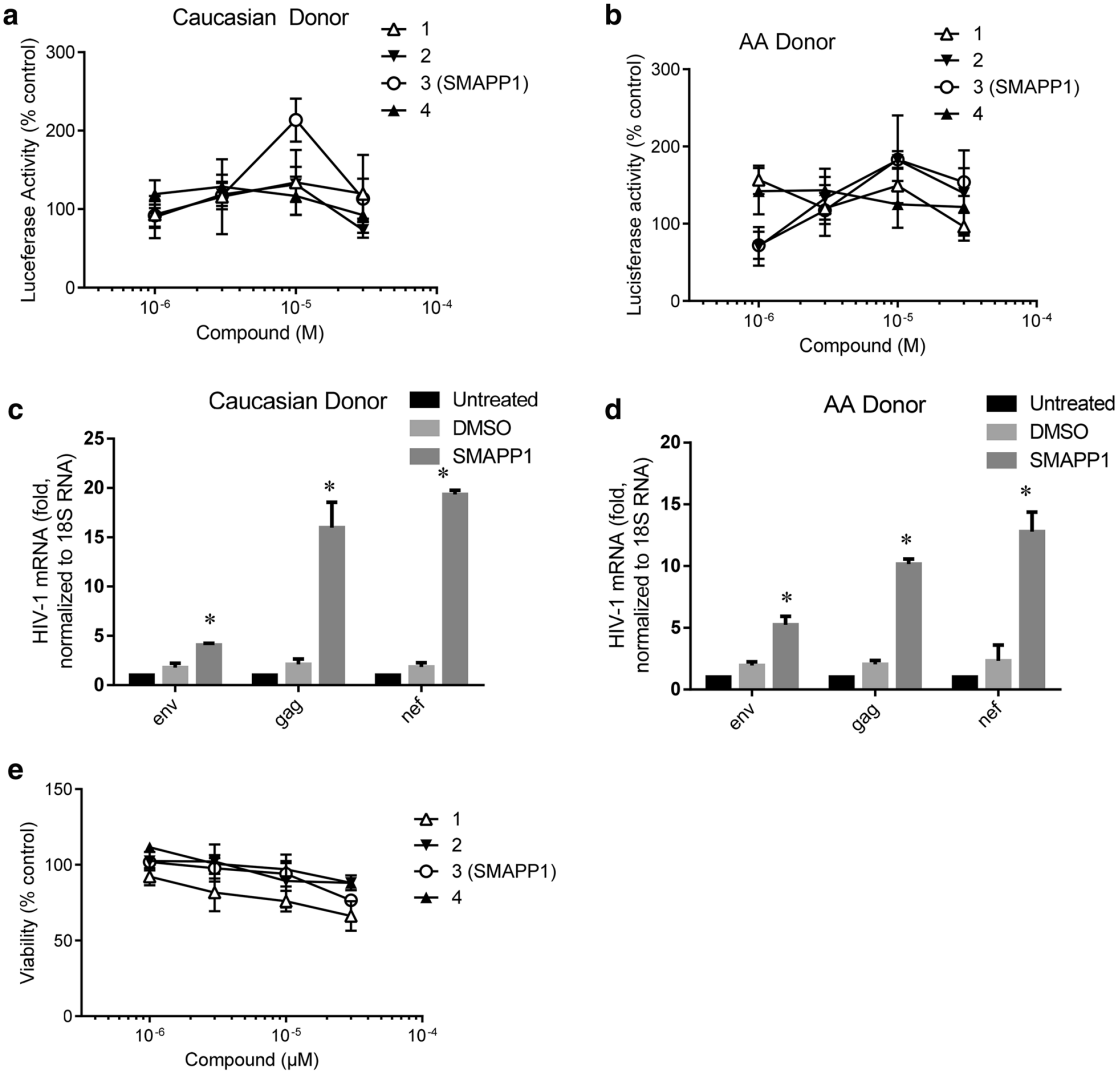
Activation of HIV-1 infection and gene expression by SMAPP1 in PBMCs. **a**, **b** Activation of one round HIV-1 infection. PBMCs obtained from a Caucasian donor (**a**) or African American donor (**b**) were stimulated with PHA and IL-2 and then infected with VSVG-pseudotyped pNL4-3.Luc.R-E- (HIV-1 Luc) virus for 18 h. After that the cells were treated for 24 h with the indicated concentrations of SMAPP1. Then the cells were lyzed and luciferase activity was measured. **c**, **d** SMAPP1 induced HIV-1 mRNA expression in PBMCs. PBMCs obtained from a Caucasian donor (**c**) or African American donor (**d**) were stimulated with PHA and IL-2 and then infected with HIV-1 Luc. The cells were left untreated, or treated with DMSO or 10 μM SMAPP1 diluted in DMSO. The cells were collected after 48 h in culture, RNA was extracted, reverse transcribed and analyzed with primers for HIV-1 *env*, *gag* and a portion of *nef* remaining in the HIV-1 Luc after the insertion of luciferase. Real-time PCR was carried on Roche 4800 using 18S RNA as a reference. The unpaired *t* test was used to determine statistical significance. *Asterisks* indicate *p* ≤ 0.05. **e** Toxicity of compounds for PBMCs. PBMCs were treated with the indicated concentrations of the compounds for 24 h. Toxicity of compounds was determined with trypan blue exclusion assay using an automated cell counter.

We next analyzed the effect of SMAPP1 on single round HIV-1 replication and cell viability in primary peripheral blood mononuclear cells (PBMCs). PBMCs obtained from two different donors, a Caucasian and an African American, were infected with HIV-1 Luc and treated with the compounds 1–4. At 10 μM concentrations, SMAPP1 showed the best activating capability in a Caucasian donor-derived cells (Figure 2a, b), whereas expression of HIV-1 Luc in PBMCs from African American donor was not significantly changed. On the other hand, treatment with SMAPP1 led to a drastic increase of HIV-1 *env*, *gag* and *nef* RNA expression in PBMCs obtained from both donors as detected by quantitative RT-PCR (Figure 2c, d), suggesting positive effect of the compound on HIV-1 transcription.

At the same time, this compound showed no significant effect on cell viability as detected by the trypan blue exclusion assay (Figure 2e). Taken together, these results indicate that SMAPP1 significantly induced HIV-1 gene expression and that the effect was due to the upregulation of viral gene transcription.

### SMAPP1 contributes to the production of HIV-1 RNA in chronically infected cell lines and quiescent primary CD4^+^ T cells

**Figure 3:**
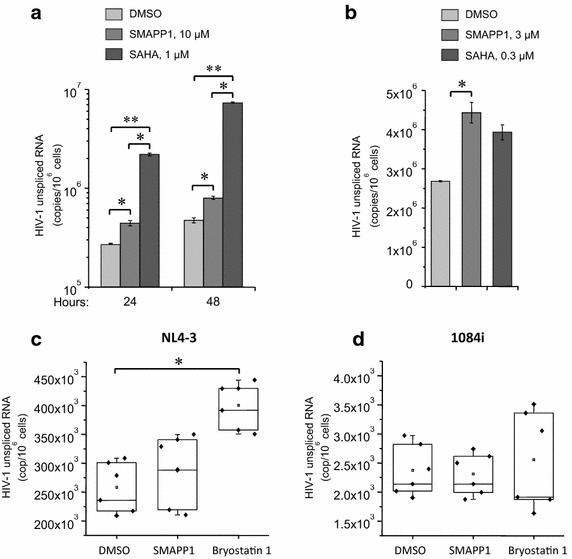
Effect of SMAPP1 on HIV-1 gene expression in chronically HIV-1 infected T cell lines and primary cells. **a** Chronically HIV-1 infected T cell line ACH-2 was pre-treated with the cocktail of antiretrovirals (lamivudine/emtricitabine, tenofovir and indinavir each in 10 µM concentrations) for 7 days, then the cells were washed and cultured in regular medium with indicated concentrations of SMAPP1 or SAHA. The cells were harvested at 24 and 48 h after the treatment, total RNA was isolated and subjected to quantitative RT-PCR analysis with the primers specific for HIV-1 *gag*. Results are shown as a mean of three independent experiments ±SD. *Asterisk* shows *p* value ≤0.05;* double asterisk* shows *p* value ≤0.01 between control cells and cells treated with SAHA. **b** The same cells were treated with a lower concentration of SMAPP1 or SAHA. Quantitative RT-PCR analysis of total RNA with oligo-dT (RT reaction) and gag-specific primers (qPCR) was performed in 24 h post-treatment. Results are shown as a mean of three independent measurements ±SD. Asterisk shows p value ≤0.01 between the control cells and cells treated with 3 µM SMAPP1. **c**, **d** Statistical analysis of the effect of SMAPP1 and bryostatin 1 on HIV-1 transcription in low-productive infected PBMCs. The PBMCs from healthy donors were activated with IL-2, infected with HIV-1 subtype B strains NL4-3 (**c**) or subtype C 1084i (**d**) (20 ng of p24 per 10 × 10^6^ cells) by spinoculation and after 8 days cultivation in medium with IL-2 were cultured for 15 days with IL-7 to transfer T cells to quiescent phase. The cultures were treated with SMAPP1 or bryostatin 1 and then cultured for 48 h. *Asterisk* shows p value ≤0.01 between the control cells and cells treated with bryostatin 1.

To test the effect of SMAPP1 on transcription of the integrated HIV-1 genome, we analyzed HIV-1 RNA production in chronically infected T lymphocytes (ACH-2) treated with this compound. As a positive control, we used a potent inhibitor of histone deacetylases (HDAC), suberoylanilide hydroxamic acid (SAHA) [35] which was recently used in preclinical trials to eliminate latent HIV-1 infection [36]. Treatment of ACH-2 cells pre-incubated with the cocktail of antiretrovirals (lamivudine/emtricitabine, tenofovir and indinavir each in 10 µM concentration) for 7 days to prevent reinfection of the cells, with 10 µM SMAPP1 led to two-fold induction of HIV-1 RNA production both at 24 and 48 h post-treatment, with the strongest effect at 48 h (Figure 3a). Although the treatment with 1 µM SAHA had a more dramatic effect showing tenfold increased HIV-1 transcription in ACH-2 cells, SAHA, unlike SMAPP1, had a marked cytotoxic effect on the treated cells (data not shown). To avoid increased cell death, associated with the treatment with toxic doses of SAHA, we analyzed the effect of clinically relevant dose 0.3 µM on HIV-1 transcription within 24 h after treatment. The dose of SMAPP1 was also threefold decreased. Data in Figure 3b indicate that both compounds displayed the similar 50–60% level of HIV-1 transcription activation.

To test the effect of PP1 activation on HIV-1 transcription in the model of latently infected primary cells, the PBMCs isolated from healthy donors were infected with HIV-1 isolate NL4-3, treated with IL-7, cultured for 15 days to transfer T cells to the quiescent stage [37], and then treated with 10 µM SMAPP1. The protein kinase C (PKC) agonist bryostatin-1 has been selected as a positive control for HIV-1 transcription activation based on its significant effect on HIV-1 activation in viral outgrowth assay [38]. Data in Figure 3c indicate that the positive effect of SMAPP1 on the viral transcription varied depending on the individual donor, but a trend of increasing HIV-1 transcription level could be observed. Bryostatin 1 at nanomolar concentrations had a significant activating effect on NL4-3 transcription in the infected PBMCs from all donors. The tested NL4-3 isolate of subtype B has been widely used as a model of HIV-1 infection in multiple experiments. To test effect of SMAPP1 on another HIV-1 subtype, we infected PBMCs from the same donors with HIV-1 subtype C isolate 1084i from Zambia, kindly provided by Dr. Charles Wood. This isolate was obtained from patient with slow disease progression and characterized by a prolonged clinically asymptomatic period (more than 4 years) [39]. Surprisingly, despite the very low basic level of the virus expression in untreated cells, neither SMAPP1 nor bryostatin 1 treatment had any statistically significant effects on the transcription (Figure 3d).

Thus, our data indicates that, SMAPP1 moderately activated HIV-1 NL4-3 transcription in chronically infected T cell lines and low-productively infected quiescent T cells. Despite the lower impact of SMAPP1 on HIV-1 transcriptional activation than the HDAC inhibitor SAHA and PKC agonist bryostatin 1, the tested PP1 targeting agent had no visible cytotoxicity at effective concentrations. Activation effect on the latent infection caused by HIV-1 subtype C isolate has not been observed.

### SMAPP1 binds to PP1 and induces its activity in vitro

**Figure 4:**
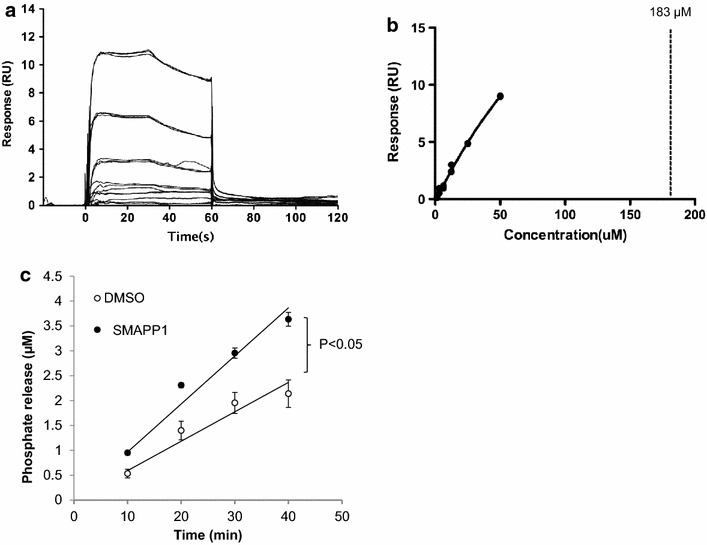
SMAPP1 binds to PP1 and upregulates PP1 activity in vitro. **a**, **b** Direct binding of SMAPP1 to recombinant PP1 protein. The direct binding interaction was measured by surface plasmon resonance using a Biacore T-200 instrument. Raw data showing binding of SMAPP1 to PP1 protein are presented in **a**. The X axis represents time in seconds and the Y axis represents changes in total mass on microchip surface, which was expressed as resonance units. A positive deflection indicated binding of SMAPP1 in solution to PP1 protein that was immobilized on a microchip surface. Each *line* represents a different concentration of SMAPP1, all between 0 and 50 μM. Each concentration was run three times. In **b**, the equilibrium dissociation constant (K_D_) was calculated based on a 1:1 binding model. The K_D_ was calculated as 183 μM for SMAPP1 binding to PP1. Each data point represents the binding level shown in **a** from different concentrations of SMAPP1 measured just before the end of the injection (~55 s time point). A *vertical dashed line* represents the small molecule concentration that results in a binding level that is 50% of the extrapolated maximal signal. Experimental data points did not reach to the actual saturation level due to limitation on available SMAPP1 concentration, which resulted in the calculation of K_D_ value from an extrapolated line. **c** SMAPP1 induces pRb-Tat dephosphorylation by PP1. Recombinant PP1α was assayed with pRb-Tat (75 μM) in the absence or presence of 200 μM SMAPP1 as indicated. The reactions were stopped at indicated time points and the phosphate release was quantified by malachite *green* assay. Initial velocity was calculated by linear regression. Unpaired t test was used to calculate p value.

We analyzed the binding of SMAPP1 to recombinant PP1 protein which was expressed in *E. coli* cells (see “Methods”), using surface plasmon resonance technology with a Biacore T-200 instrument (Figure 4a, b). PP1 was immobilized on a sensor chip and different concentrations of SMAPP1 were injected over the surface of the chip. Direct binding of the compound to PP1 was measured in real time and binding affinity was calculated based on a 1:1 binding model. SMAPP1 bound to PP1 with a Kd value of 183 μM, showing its ability to interact with PP1 in vitro (Figure 4a, b).

We previously used hybrid peptides containing a retinoblastoma protein-derived phosphopeptide linked to an RVxF-containing sequences derived from HIV-1 Tat as substrates for PP1 for the analysis of the effect of PP1-targeting small molecules [30]. Here we analyzed the effect of SMAPP1 on dephosphorylation of the pRb-Tat peptide (HIPR(pS)PYKFPSSPLR KKCCFHCQVCFITK). Addition of SMAPP1 at ~3-fold molar excess (200 µM) over pRb-Tat (75 µM) induced pRb-Tat dephosphorylation and increased the rate of dephosphorylation (Figure 4c).

Thus, Biacore experiments demonstrated a direct interaction between SMAPP1 and PP1 and enzymatic assays showed that PP1 activity was increased in the presence of SMAPP1.

### SMAPP1 induces CDK9 phosphorylation

**Figure 5:**
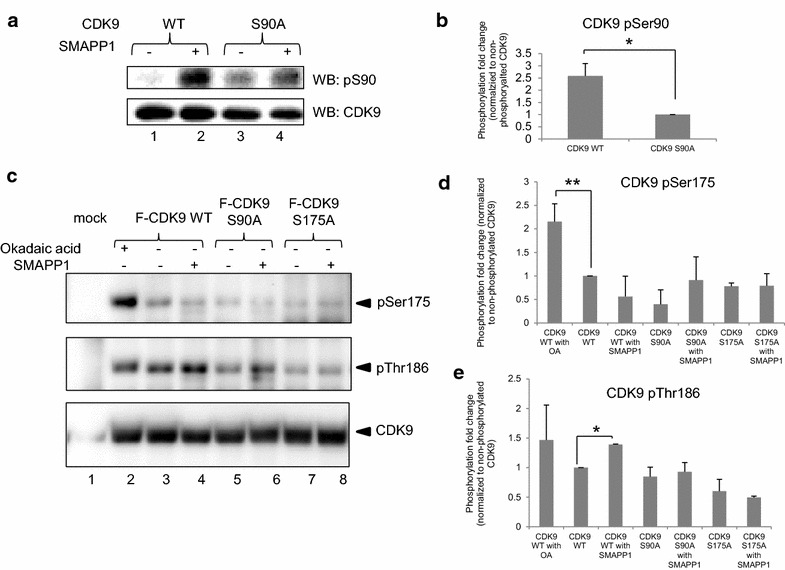
Effect of SMAPP1 on CDK9 phosphorylation. **a** SMAPP1 induces CDK9 Ser90 phosphorylation. 293T cells were transfected with vectors expressing Flag-CDK9 WT (*lanes 1–2*) or Flag-CDK9 S90A mutant (*lanes 3–4*) and after 48 h in culture treated with 10 μM SMAPP1 for 24 h. CDK9 was immunoprecipitated from cellular lysates with anti-Flag antibodies and analyzed by immunoblotting with anti-CDK9 antibodies and Ser90 phospho-specific antibodies. **b** Quantification of four independent experiments similar to the one shown in **a**. *Asterisk* shows p value ≤0.05. **c** Effect of SMAPP1 on CDK9 Ser175 and Thr186 phosphorylation. 293T cells were transfected with vectors expressing Flag-CDK9 WT (*lanes 2–4*) or Flag-CDK9 S90A mutant (*lanes 5–6*) or Flag-CDK9 S175A (*lanes 7–8*) and after 48 h in culture treated where indicated with 10 μM SMAPP1 for 24 h or 0.1 μM okadaic acid for 2 h. *Lane 1* mock transfection. CDK9 was immunoprecipitated from cellular lysates with anti-Flag antibodies and analyzed by immunoblotting with anti-CDK9 antibodies, Ser175 phospho-specific and Thr186 phospho-specific antibodies. **d**, **e** Quantification of two independent experiments. *Asterisk* shows p value ≤0.05 and *double asterisk* shows p value ≤0.01 between the indicated *bars*.

We previously identified serine amino acid residue 90 (Ser90) of CDK9 as CDK2 phosphorylation site and showed that its phosphorylation induces HIV-1 transcription (reviewed in [11]). We also previously showed that okadaic acid, a general inhibitor of PP1 and PP2A, induced CDK9’s Ser 175 phosphorylation [22] and that expression of PP1 inhibitory peptide, cdNIPP1, induced CDK9’s Thr 186 phosphorylation [29]. To analyze the effect of SMAPP1 on CDK9 phosphorylation on Ser90, Ser175 and Thr186, CDK9 WT and indicated mutants were expressed in 293T cells, the cells were treated with 10 μM SMAPP1 or with DMSO, and CDK9 was immunoprecipitated and analyzed by immunoblotting with phospho-specific antibodies or CDK9-specific antibodies [25]. CDK9 Ser90 phosphorylation was increased in the SMAPP1-treated cells (Figure 5a, lane 2). The specificity of the antibodies against CDK9 was confirmed with the CDK9 S90A mutant, which showed reduced phosphorylation of the corresponding residue (Figure 5a, lanes 3 and 4, Figure 5b). We next analyzed phosphorylation of CDK9 Ser 175 using phospho-specific antibodies obtained from Dr. Jonathan Karn and Thr186 phosphorylation using commercially available antibodies. While okadaic acid induced CDK9 Ser 175 phosphorylation (Figure 5c, d), there was no effect of SMAPP1 (Figure 5c, d). However, SMAPP1 treatment produced a small but statistically significant increase in CDK9 Thr186 phosphorylation (Figure 5c, e). Hence, SMAPP1 selectively induces phosphorylation at Ser90 and Thr186 residues of CDK9.

### Effect of SMAPP1 on cellular proteome

**Figure 6:**
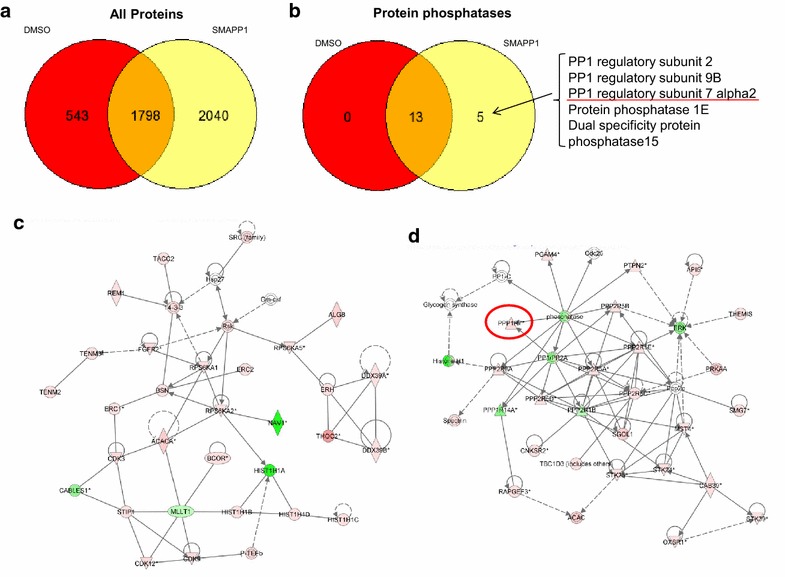
Effect of SMAPP1 on cellular protein expression. **a** Venn diagram of proteins from CEM T cell proteins treated with 10 μM SMAPP1 or DMSO as a control. Cellular proteins were extracted, trypsinized and tryptic peptides were separated by ion exchange chromatography and then analyzed by high resolution mass spectrometry. Proteins were identified by Proteome Discoverer 2.1. Proteins eluted in 0, 25, 50, 100, 250 and 500 NaCl were combined. **b** Venn diagram of protein phosphatase-related proteins identified in CEM T cells. **c**, **d** Peptides detected in 100 mM salt fraction were quantified using SIEVE 2.1 and exported to Ingenuity software for the analysis of protein networks. Transcription (**c**) and protein phosphatase (**d**) networks are shown. Up-regulated genes are colored in *red* and down-regulated genes are colored in *green*.

**Figure 7:**
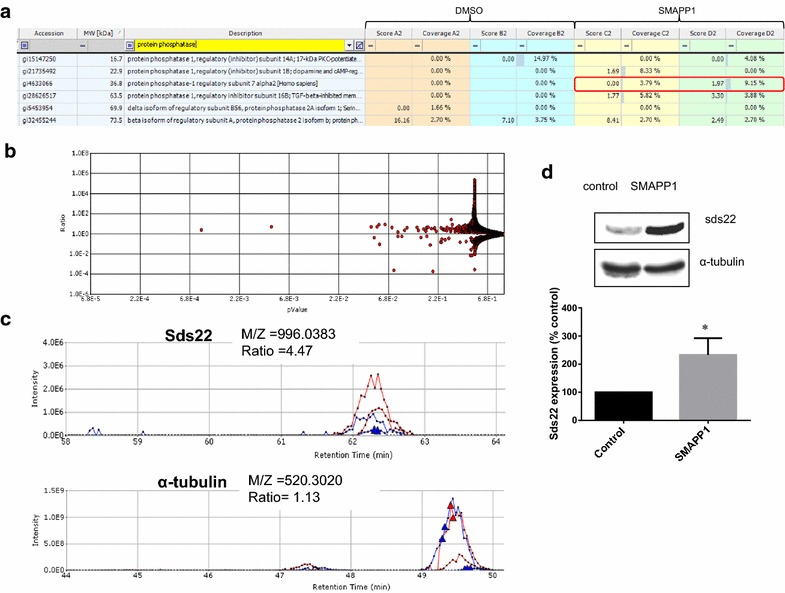
Induction of Sds22 expression by SMAPP1. **a** Proteome Discoverer analysis of Sds22 expression. CEM T cells were treated with 10 μM SMAPP1 or DMSO as control and proteins were analyzed by mass spectrometry. Proteins containing “protein phosphatase” query identified in Proteome Discoverer 2.1 are shown. Sds22 is indicated in *red*. **b** Quantitative analysis by SIEVE 2.1. The *volcano plot* shows ratios of peptides present in 100 mM salt fraction in SMAPP1 versus DMSO treated samples with corresponding p values. **c** Quantitative analysis of Sds22 expression using SIEVE 2.1. Ion elution profiles are shown in *blue* for control samples and in *red* for the CEM T cells treated with SMAPP1. *Triangles* indicate the time points at which MS/MS was conducted. The *upper panel* shows the elution of the Sds22-derived ion. The *lower panel* shows the elution of the α-tubulin-derived ion. Results from two independent experiments are shown. Integration of the peaks was performed by SIEVE 2.1 and the ratios of the ion peaks in SMAPP1 versus control cells are shown. **d** Validation of Sds22 expression by Western blotting. CEM T cells were treated with 5 µM of SMAPP1 overnight. Cell lysates were resolved on 10% SDS-PAGE and immunoblotted with antibodies against Sds22 or α-tubulin as loading control. Four separate experiments were conducted and quantified as shown in the *lower panel*. *Asterisks* indicate p < 0.05.

To determine whether SMAPP1 has a global effect on protein expression in T cells, we conducted proteomic analysis of SMAPP1-treated CEM T cells using a recently developed protocol in which tryptic peptides from total cell lysates were fractionated on a cation exchange column [30]. In total, we detected over 2,000 proteins in the DMSO and over 3,000 proteins in SMAPP1-treated cells with 1,798 proteins detected in both conditions (Figure 6a). Proteins related to protein phosphatases were upregulated in SMAPP1-treated cells including PP1 regulatory subunit 7 or Sds22 (Figure 6b). We used quantitative label-free analysis of differential peptide expression using SIVE 2.1 software for the proteins eluted in 50 mM NaCl, which contained PP1-realted proteins and which showed majority of the peptides being equally expressed in DMSO and SMAPP1 treated cells (see below). Pathway analysis was performed using Ingenuity software and showed several protein networks with upregulated protein expression including transcription and protein phosphatases networks (Figure 6c, d). Of note, in the transcription network, the level CDK9 was upregulated (Figure 6c). In the protein phosphatases network, both PP1 and PP2A were upregulated as well as number of PP2A and PP1 regulatory subunits including PP1 regulatory subunit 7 (Sds22) and 14A (Figure 6d). We further analyzed the expression of PP1 regulatory subunit 7 (Sds22) and subunit 14A using Proteome Discoverer 2.1, and the results showed the presence of Sds22-derived peptides only in the SMAPP1 treated cells whereas subunit 14A was detected in both DMSO and SMAPP1 treated cells (Figure 7a). Expression of Sds22 was quantified by SIEVE 2.1 that allowed extraction and quantification of ions from the 50 mM NaCl fraction and which showed the majority of the peptides being equally expressed in DMSO and SMAPP1 treated cells (Figure 7b). Quantification of Sds22-related peptide showed 4.5-fold increased expression in the cells treated with SMAPP1 (Figure 7c). In contrast, expression of α-tubulin was similar in DMSO and SMAPP1-treated cells (Figure 7c). The increased Sds22 expression was confirmed by immunoblotting analysis with anti-Sds22 specific antibodies (Figure 7d). While we do not yet know the molecule mechanism of this upregulation, our recent study showed that partial siRNA-mediated knockdown of PP1α led to the increased expression of PP1 regulatory subunit, SIPP1, likely as a compensatory cell response [40]. Thus, increased expression of Sds22 could also be a cellular compensatory response to PP1 deregulation or its partial cellular inhibition.

### In silico analysis of SMAPP1 and PP1 binding

**Figure 8:**
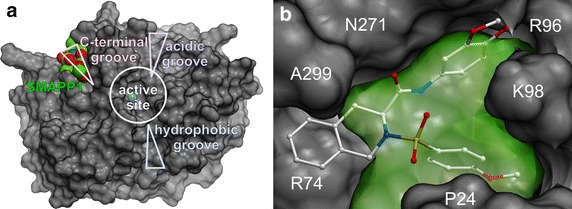
SMAPP1 binds to the C-terminal groove of PP1. **a** Model of SMAPP1 binding to PP1. A model was constructed by docking analysis with ICM software. **b** Predicted binding position of SMAPP1 on the PP1α surface. Protein surface is shown in *gray* color, cavity #1 (see Table 1) is shown in *transparent green* color. Several protein residues are *labeled* for clarity.

To further analyze the effect of SMAPP1 on PP1, we conducted an in silico docking analysis of the molecule on the surface of PP1 using ICM software and coordinates of the PP1 crystal structure. Molecular model of PP1α was built with ICM-Pro software package using PDB structure 3E7A [41]. SMAPP1 was docked to top 10 predicted binding sites on the surface of PP1 (Table 1). The top docking site with the lowest ICM score of -30.2 is shown in Figure 8a (overall view) and Figure 8b, which shows details of the interaction.

**Table 1:** Prediction for SMAPP1 binding to PP1α using ICM docking algorithm

#	Docking site (PP1 residue number)	Cavity volume, Å^3^	Best ICM score
1	20–22, 24–25, 67–71, 73–74, 77, 96–99, 270–274, 299	334.5	−30.2
2	208–211, 218–221, 226, 246, 249, 251, 256–258, 263, 265	195.5	−20.1
3	64, 66, 92, 96, 124–125, 130, 134, 206, 221, 248–250, 267, 272	198.4	−5.6
4	49–50, 53–56, 59, 86, 116:117, 119	116.6	−18.2
5	47, 49–52, 187:191	121.6	−22.9
6	176–178, 180–181, 216, 231, 234–235, 238	129.9	−16.0
7	56–60, 84–86, 284–286	57.4	−21.3
8	197–198, 218–219, 222–225	78.3	−18.2
9	211, 229, 257–261	56.3	−22.6
10	124, 127, 129–130, 195–197, 202, 206, 223	72.1	−18.6

Taken together, our results indicated that we have identified a small molecule compound, SMAPP1, which can induce HIV-1 transcription and reactivate latent HIV-1 provirus. This compound increases CDK9 phosphorylation on several key residues and activates PP1 in vitro and also induces Sds22 expression in the treated T cells.

## Discussion

In the present study, we extended our previous findings of the small molecule mimetics of the “RVxF”-docking peptide by identifying compounds that activate HIV-1. We previously identified HIV-1 inhibitory 1H4 compound [30] and, more recently, 1E7-03 compound [31]. Both molecules disrupt the interaction of HIV-1 Tat’s RVxF sequence with PP1 in vitro and inhibit HIV-1 transcription as well as replication in cultured cells [31]. In the current study, we have developed a novel library of sulfonamide-containing compounds and, out of this library, identified a novel compound SMAPP1 which is able to induce HIV-1 transcription and replication with no observed toxicity.

We used several cell-based models of HIV-1 latency to test the effect of PP1-targeting small molecule compounds on the activation of viral expression. These include one round HIV-1 infection, latently infected Jurkat (lymphoblastoid T cells), THP (monocytes) cell lines, chronically infected CEM T cells (ACH-2) and primary CD4+ T cells within the pool of infected PBMCs treated with IL-7 to transfer them to quiescent phase. While SMAPP1 induced HIV-1 in chronically and latently infected T cell line two to threefold, in PBMCs we could not detect significant effect on total HIV-1 transcription, whereas a trend to transcription reactivation, especially in the case of HIV-1 subtype B infection, was observed. On the other hand, analysis of HIV-1 gene expression in acutely-infected PBMCs from various donors, treated with SMAPP1, showed increased transcription of HIV-1 mRNA; the level of both unspliced and single-spliced mRNA species was detected. High variability of SMAPP1 effect on HIV-1 transcription in the cells from different donors, as well as lack of visible effect on the transcription of HIV-1 subtype C virus genome within the low-productive infection in primary cells suggests that impact of this compound on HIV-1 infection depends on the nature of infected cells and probably the virus subtype.

Our previous studies showed that HIV-1 Tat interacts with PP1 and translocates it to the nucleus [42]. PP1-targeting HIV-1 inhibitory compound 1E7-03 prevents interaction of Tat with PP1 and nuclear targeting of PP1 [31]. CDK9 was shown to be phosphorylated on T-loop residues, Ser175 and Thr186, and also on Ser90 located within a loop which is adjacent to the T-loop (reviewed in [11]). The CDK9 Thr186 phosphorylation is required for the enzymatic activity of CDK9 and also facilitates the association of CDK9/cyclin T1 with 7SK RNA snRNP [20, 21]. CDK9 Thr186 is phosphorylated by CDK7/cyclin H [43]. CDK7/cyclin H has now emerged as a universal CDK-activating kinase that phosphorylates and regulates cell-cycle-related CDKs, such as CDK1, 2 and 4, and also CDKs involved in transcriptional regulation such as CDK8, 9, 12 and 13 (see for details [44]). We showed that inhibition of PP1 increased phosphorylation of CDK9 Ser175 residue, decreased CDK9 activity and reduced RNA polymerase II phosphorylation in vivo [22]. Recently, reduced CDK9 Ser175 phosphorylation was shown to enhance HIV-1 transcription by preventing CDK9/cyclin T1 interaction with Brd4 making CDK9/cyclin T1 available for recruitment by HIV-1 Tat protein [24]. However, in activated T cells CDK9 Ser175 was phosphorylated, and this phosphorylation increased the affinity of CDK9/cyclin T1 to Tat [24]. We also recently showed that CDK9 Ser90 is phosphorylated by CDK2 and that this phosphorylation induces HIV-1 transcription [25]. Analysis of CDK9 phosphorylation in the cells treated with SMAPP1 conducted here showed increased Ser90 phosphorylation suggesting that PP1 might either directly control CDK9 Ser90 phosphorylation or have an indirect effect on CDK2 activity. In vitro, we observed dephosphorylation of CDK9 by PP1 when CDK9 was phosphorylated by CDK2 (Ammosova and Nekhai, unpublished) suggesting that PP1 may also dephosphorylate CDK9 Ser90.

We analyzed the effect of SMAPP1 on PP1 in vitro, using a Biacore assay with Chip-immobilized bacterially expressed PP1. While we observed relatively weak binding with Kd = 183 μM, the presence of SMAPP1 induced dephosphorylation of a peptide substrate containing an RVxF motif suggested that it may interfere with the binding of RVxF motif or some other sites where the hybrid substrate interacts with PP1. The latter is further supported by the in silico docking analysis, which showed preferred interaction of SMAPP1 with the C-terminal groove of PP1, which may also be involved in the interaction of the peptide substrate with PP1. Analysis of proteins expressed in T cell treated with SMAPP1 showed upregulation of the PP1-regulatory subunit, Sds22. Expression of Sds22 was also verified by immunoblotting analysis. Sds22 is evolutionarily conserved ancient interactor of PP1 that along with Inhibitor 3 forms a complex with PP1 and helps to translocate it to the nucleus [45]. Sds22 also converts PP1 to inactive form [45]. Thus, over expression of Sds22 may change the cellular distribution of PP1 and potentially deregulate cellular PP1 or reduce PP1 availability for Tat recruitment and CDK9 dephosphorylation. While the crystal structure of PP1 in complex with Sds22 is yet to be determined, biochemical studies showed that the N-terminal part of the PP1 C-terminal groove might interact with Sds22 [46]. Thus SMAPP1 can potentially interfere with the binding of Sds22 to PP1 leading to the upregulation of Sds22 expression as a cellular compensatory response.

Our study shows that HIV-1 can be induced by a small molecular compound that interferes with PP1 [1]. These results are significant in light of the current therapy named the kick-and-kill approach where HDAC inhibitors are used to activate the virus followed by combination antiretroviral therapy (cART). Current ideas to use HDAC inhibitors for virus reactivation are promising since this method provides four to fivefold increase in viral replication [47]. The cART is efficient in eradicating the circulating virus in plasma by inhibition of productive infection. However, the current drug compositions are not able to completely eliminate the virus [48], since HIV-1 is capable of maintaining latent infection in stable reservoirs such as resting CD4+ T cells, naive T cells and CD34+ multipotent hematopoietic stem cells [49, 50]. Furthermore, interruption of the therapy leads to the rapid increase of viral population [51, 52], whereas a long combinational cART treatment has negative side effects, including fatigue, diarrhea [53], neurocognitive abnormalities, cardiovascular diseases [54–56]. Here, we show that SMAPP1 is the first example of small molecule targeting PP1 that induces HIV-1 transcription and potentially could be used as the latency reversing agents in patients on cART treatment. Combination of the latency reversing drugs such as PKC agonist bryostatin showed improved HIV-1 activation with the reduction of toxicity [57]. We are currently testing a synergy of SMAPP1 with SAHA and other HDAC inhibitors. Taken together, our study points to PP1 as a new drug target for novel antiretroviral therapeutics. These therapeutics can be aimed at reversing HIV-1 latency as we show here with SMAPP1 which will have to be used with cART to prevent virus rebound. Alternatively, PP1 can be targeted to cure HIV-1 with PP1-targeting HIV-1 inhibitors such as 1E7-03 [58] which can be used alone or in combination with cART.

## Methods

Cells were obtained from ATCC (Manassas, VA, USA). pNL4-3.Luc.R^−^E^−^ (Gervaix et al. 1997), as well as latently-infected CEM subclone ACH-2 [59] and chronically-infected subclone of promyelocytic HL-60 cell line OM10.1 [60] were obtained from the NIH AIDS Research and Reference Reagent Program. PBMCs were purchased from Astarte Biologics (Redmond, WA, USA). Anti-Flag antibodies and anti-tubulin antibodies were from Sigma (Atlanta, GA, USA). Protein G agarose was from Upstate (Lake Placid, NY, USA). Antibodies against PP1α were from EMD Chemicals (Gibbstown, NJ, USA). Anti-GFP and anti-CDK9 antibodies were from Santa Cruz Biotechnology (Dallas, TX, USA). CDK9 Thr186 phospho specific antibodies were from Cell Signaling Technology (Beverly, MA, USA). CDK9 Ser175 phospho specific antibodies were a gift from Dr. Jonathan Karn (Case Western Reserve University). CDK9 Ser90 phospho specific antibodies were prepared by Dr. Monique Beullens (Catholic University of Leuven, Belgium) as previously described [25].

### Design of the 1E7-03 derivatives library

To generate novel compounds with alternative chemical scaffolds, we conducted a pharmacophore search from Enamine stock collection followed by visual selection. A phase program from the Schrodinger suite was used for screening. Pharmacophore model was constructed using atoms of the aromatic groups and ester oxygen at position 9 of the acridine core. Putative ligands were considered for those which matched at least 3 pharmacophore points (out of 5) with the acridine pharmacophore model. The preference was given to sulfonamides enriched with aromatic groups. An acridine series with the amide group instead of ester in position 9 of the acridine were prepared at Enamine facilities. In total, 38 compounds were included into this study.

### Single round HIV-1 replication assay

CEM T cells or PBMCs were infected with VSVG-pseudotyped pNL4-3.Luc.R-E-virus (HIV-1 Luc) prepared as previously described [32]. PBMCs were stimulated by treating with 2.5 μg/mL phytohemaggultinin (PHA) for 24 h and then activated for another 24 h with 10 units/mL IL-2 before the infection with HIV-1 Luc. PBMCs were infected with HIV-1 Luc, cultured at 0.5 × 10^6^ cells/mL in 6-well plates at 37°C and 5% CO_2_ for 24 h and then treated with indicated concentrations of compounds. The cells were collected after 48 h in culture, washed with PBS and resuspended in 100 μL of PBS. Then, 100 μL of reconstituted luciferase buffer (Luclite Kit, Perkin Elmer) was added to each well and after 10 min incubation. The lysates were transferred into white plates (Perkin Elmer) and luminescence measured using Labsystems Luminoscan RT equipment (Perkin Elmer).

### Cell viability assays

CEM T cells or PBMCs were cultured as described above in 96-well plates at 37°C. Cell viability was determined by trypan blue assay using TC-10 automated cell counter (Bio-Rad).

### HIV-1 activation in ACH-2 cells

The cells were plated in 6-well plates at a concentration of 10^6^ cells/mL in RPMI. The cells were then incubated with varying doses of compounds for 36–48 h. Cells were harvested from the wells by scraping and washed three times with PBS. Luciferase levels in the cells were assessed using a commercial kit (Promega; Madison, WI, USA). Briefly, the cells were lysed for 30 min at room temperature with passive lysis buffer and centrifuged at 10,000×*g* for 2 min. Then 10 µl of samples were added to individual wells, followed by 70 µl luciferase substrate/assay buffer. Samples were tested in triplicates. Luminescence was measured in a Veritas Microplate Luminometer (Turner Biosystems).

### HIV-1 activation in chronically infected cell lines

Chronically HIV-1 infected cell lines ACH-2 cells were maintained at 37°C and 5% CO_2_ in 25 and 75 cm^2^ tissue culture flasks with RPMI-1640 culture medium supplemented with 10% Fetal Bovine Serum, penicillin/streptomycin (100 μg/mL), and l-Glutamine (lymphoid and myeloid cells). Before experiments, the cells were incubated for 7 days with the cocktail of four antiretroviral drugs (Emtricitabine, Tenofovir, Indinavir, and Lamivudine), each in concentration 10 µM. Then the cells were washed with PBS and cultured in regular media, with various concentrations of solutions of SMAPP1 or SAHA diluted in DMSO. The control cells were treated with equivalent volumes of DMSO. The cells were harvested at 24 and 48 h post-treatment, the total RNA was isolated and used for quantitative RT-PCR analysis.

### Establishment of latently HIV-1 infected primary CD4^+^ T cells

Briefly, the primary CD4+ T-cells were isolated from either PBMCs (peripheral blood mononuclear cells), activated using a-CD3/CD28 antibodies and infected with HIV-dNef-IRES-GFP virus. The pure population of HIV infected cells (GFP expressing) was purified by FACS sorting and further expanded with a-CD3/CD28 antibodies. Once cell population reached between 50 to 100 × 10^6^, the cells were placed on feeder cells in the presence of IL-2 to allow proviral latency establishment. Usually, after 6 weeks most of the cells enter into a quiescent state, characterized by loss of GFP expression, the cessation of DNA synthesis and a huge reduction in cell size [61]. Cell characterizations demonstrate that majority have a central memory phenotype. Very few of the silenced cells have lost the provirus, since more than 95% could be efficiently reactivated via T cell receptor stimulation.

### Model of latent infection with HIV-1 in PBMCs

Total PBMCs from healthy seronegative donors were activated with 50 U/mL IL-2 and cultured for 4 days. Then the cells were infected with a replication competent HIV-1 strains NL4-3 (subtype B) or 1084i (subtype C) by spinoculation at 1,200×*g* for 2 h at room temperature. Infected cultures were expanded to 8 days in medium containing 50 U/mL IL-2. When the infected culture contained 10–15% infected cells as determined by qPCR, the cultures were placed in a quiescent phase for 15 days by cultivation in the medium containing 1 ng/mL IL-7. At the end of the 15 days resting phase, the cultures were treated with SMAPP1 compound or control drugs and then cultured for 24 or 48 h.

### RNA isolation and quantitative RT-PCR

For quantitative analysis of HIV-1 RNA, total RNA was purified from the lysates of chronically HIV-1 infected cell lines ACH-2 and OM-10.1. RNA was isolated using Trizol Reagent (Invitrogen, Carlsbad, CA) according to the manufacturer’s protocol. A total of 0.5 μg of RNA from the RNA fraction was treated with 0.25 mg/mL DNase I RNase-free (Roche, Mannheim, Germany) for 60 min in the presence of 5 mM MgCl_2_, followed by heat inactivation at 65°C for 15 min. A 200–250 ng aliquot of total RNA was used to generate cDNA with the GoScript Reverse Transcription System (Promega, Madison, WI, USA) using oligo-dT reverse primers. Subsequent quantitative real-time PCR analysis was performed with 2 µl of undiluted and 10^−1^, and 10^−2^ diluted aliquots of RT reaction mixes. The iQ SYBR Green Supermix (Bio-Rad, Hercules, CA, USA) was used with the primers specific for HIV-1 gag gene: Gag1483-F (5′-AAGGGGAAGTGACATAGCAG-3′) and Gag1625-R (5′-GCTGGTAGGGCTATACATTCTTAC-3′) amplifying 143 nucleotide fragment of HIV-1 gag gene. Serial dilutions of DNA from 8E5 cells (a CEM T cell line containing a single copy of HIV-1 LAV provirus per cell) were used as the quantitative standards. To normalize HIV-1 RNA quantifications in the human cells to the target cell DNA, the β-globin gene was quantified by real-time PCR using a set of β-globin-specific primers: BGF1: 5′-CAACCTCAAACAGACACCATGG-3′), BGR1: 5′-TCCACGTTCACCTTGCCC-3′ and probe BGX1: 5′-FAM-CTCCTGAGGAGAAGTCTGCCGTTACTGCC-TAMRA-3′. Real-time PCR reactions were carried out at least in triplicate using the PTC-200 Peltier Thermal Cycler with Chromo4 Continuous Fluorescence Detector (both from MJ Research) and Opticon Monitor 2.03 software.

### Transfections

293T cells were seeded in 6 well plates to achieve 50% confluence on the day of transfection. The cells were transfected with the indicated plasmids using Lipofectamine Plus reagent (Life Technologies) following the manufacturer’s protocol. The efficiency of transfection was verified using a plasmid encoding green fluorescent protein. The cells were cultured for 48 h post-transfection and then analyzed for phosphorylation of CDK9.

### Immunoprecipitations

293T cells were lysed in whole cell lysis buffer (50 mM Tris–HCl, pH 7.5, 0.5 M NaCl, 1% NP-40, 0.1% SDS) supplemented with protease cocktail. CDK9 was precipitated with anti-Flag antibodies as we previously described [25]. Briefly, 400 μg of lysate and 800 ng of antibodies combined with 50 μL of 50% slurry of protein A/G agarose were incubated for 2 h at 4°C in a TNN Buffer (50 mM Tris–HCl, pH 7.5, 150 mM NaCl and 1% NP-40). The agarose beads were precipitated, washed with TNN buffer, resolved in 10% Tris–Glycine SDS-PAGE, transferred to polyvinylidene fluoride (PVDF) membranes and immunoblotted with appropriate antibodies.

### Western blot analysis

Whole cell lysates of CEM cells treated with SMAPP1 were prepared by the addition of 1X SDS and the samples were heated for 5 min at 95°C. The samples were then resolved in 10% SDS-PAGE and transferred to a PVDF membrane. The membrane was blocked with 5% milk in PBS supplemented with 0.1% Tween-20 and probed with Sds22 and α-tubulin antibodies. Images were developed on ChemiDoc™ XRS + System (Bio-Rad).

### Sample preparation for mass spectrometry analysis

CEM T cells were collected and lysed with whole cell lysis buffer (50 mM Tris HCl, 500 mM NaCl, 1% NP40, 0.1% SDS) that was supplemented with protease inhibitors. The insoluble nuclear material was removed by centrifugation for 20 min at 21,000×*g*. The supernatant was collected and protein concentration was measured using the BCA protein assay. Proteins were precipitated with cold acetone (4× the volume of the cell lysate volume) and incubated at −20°C for 30 min. The samples were then centrifuged at 13,000×*g* for 10 min and the supernatant was discarded. The pellets were dried for 10 min at room temperature and then resuspended in 100 μL of 100 mM ammonium bicarbonate buffer containing 10 mM DTT. The samples were heated at 95°C for 5 min to be reduced. The samples were then alkylated with 15 mM iodoacetamide in the dark for 20 min at room temperature. Trypsin gold was then added and the samples were incubated overnight at 37°C. A 100 mg C18 solid phase cartridge (Discovery, Supleco) was activated with 1 mL of methanol. The column was then equilibrated with 0.046% of trifluoroacetic acid. Trypsin hydrolyzates were passed through the column, which was then washed with 0.046% trifluoroacetic acid and the samples were eluted with 80% acetonitrile containing 0.046% of trifluoroacetic acid. A column was prepared by cutting a small piece of glass fiber (Applied Biosystems) with a 1 mL micropipette tip that has been clipped. The glass fiber was fed into a 200 μL pipette tip by a 1 mL pipette with a clipped tip and 20% SCX resin (POROS 50 HS, Perspective Biosystems) was added to the pipette tip. The column was equilibrated with 100 μL of 0.5% formic acid in 0.25% acetonitrile (equilibration buffer). Sodium chloride (NaCl) solutions (concentrations 25–500 mM) were prepared in equilibration buffer. The samples were loaded in the column and washed twice with 100 μL equilibration buffer. The samples were then collected with the varying concentrations of NaCl solution and then dried again in a Speed-Vac centrifuge (Savant).

### Mass spectrometry and data analysis

The mass spectra of the peptides were detected with a data-dependent 4-event scan method (a survey FT-MS parent scans followed by sequential data-dependent FT-MS/MS scans on the three most abundant peptide ions from the parent scan). Protein identifications were carried out with Proteome Discoverer 1.2 software using the SEQUEST search engine for protein database searching and using the International Protein Index (IPI) Human Protein Database (version 1.79). A sequential database search was performed using human FASTA database. Only peptides having cross-correlation (XCorr) cutoffs of 2.6 for [M + 2H]2+, 3.0 for [M + 3H]3+ and higher charge state were considered. These SEQUEST criteria thresholds typically result in a 1–2% of False Discovery Rate. FDR was determined by searching on a decoy database. We used SIEVE 2.1 software (Thermo) for label-free quantitative analysis of the high resolution MS spectra produced by Orbitrap MS scans. We also explored protein networks in SMAPP1-treated cells by uploading the results of SIEVE 2.1 analysis into Ingenuity (Qiagen) and performing pathway analysis.

### Expression of recombinant PP1

BL21 (DE3) *E. coli* cells (Invitrogen) were co-transformed with al vector RP1B, which expresses human PP1α (residues 7-300), and pGR07, which expresses GroEL/GroED chaperones (both gifts from Dr. Mathieu Bollen and Monique Beullens, KULeuven, Belgium). The cells were grown in media supplemented with 1 mM MnCl_2_ at 30°C to an A_600_ ~ 0.5. Then arabinose (2 g/L) was added to induce expression of the GroEL/GroES chaperones. When A_600_ ~ 1 was reached, the cells were transferred to 10°C and PP1 expression was induced with 0.1 mM IPTG for 20 h. Harvested cells were lysed using sonication in a solution containing in 50 mM Tris–HCl (pH 8.0), 5 mM imidazole, 700 mM NaCl, 1 mM MnCl_2_, 0.1% Triton X-100 (v/v) and protease inhibitors. His-tagged PP1 was purified using a Ni–NTA IMAC column (Qiagen). PP1 was then dialyzed and stored at −70°C in 50 mM Tris–HCl (pH 8.0), 5 mM imidazole, 700 mM NaCl, and 1 mM MnCl_2_. PP1 activity was then assayed as previously described [30].

### Surface plasmon resonance (SPR)

All SPR measurements were conducted on a Biacore T200 instrument (GE Healthcare, Piscataway, NJ, USA) at 25°C. Recombinant His-tagged PP1 was immobilized on a Ni–NTA sensor chip (GE Healthcare). The two flow cells of the sensor chip were primed with running buffer (0.01 M HEPES pH 7.4, 0.15 M NaCl, 0.005% v/v Surfactant P20, 1% DMSO and 2 mM MnCl_2_) and loaded with 0.5 mM NiCl_2_ at a flow rate of 10 μL/min for 180 s. After NiCl_2_ injection, both flow cells were washed twice with the running buffer at flow rate of 100 μL/min for 40 s. PP1 was diluted with the running buffer to a concentration of 200 nM and was then passed over the second flow cell of the sensor chip at a flow rate of 5 μL/min. The final amount of PP1 immobilized on the surface was 3,500 RU. The first flow cell was only loaded with Ni^2+^ and was used as a reference for non-specific background binding during the experiment. For binding and kinetics experiments, all compounds were diluted in the running buffer, at 50, 25, 12.5, 6.25, 3.125, 1.56 and 0 μM and were passed over the two flow cells at a flow rate of 100 μL/min for 60 s. The number of response units was recorded after the subtraction of the reference flow cell’s value (Fc2-1). Three repetitions were performed for each injection. Data were analyzed using the BiaEvaluation software of Biacore with a 1:1 binding model.

### Molecular modeling

All molecular modeling was performed using ICM-Pro software package v. 3.8-1 (Molsoft LLC, USA). 3D atomic structures of PP1α were taken from the PDB database (PDB IDs: 1FJM, 3E7A, 3E7B, 3EGG, 3EGH, 3HVQ, 3 N5U). Preliminary analysis showed high similarity of these structures (mean pairwise RMSD of Cα atoms of the main protein chain was below 0.5 Å). Structure 3E7A [41] with the best resolution of 1.6 Å was selected to build the molecular model of PP1α. The 2,400 hydrogen atoms were added and conformations of the side chains were locally optimized. The amino acid residues were renumbered in according to the sequence PP1A_HUMAN (UniProt ID: P62136). Cavities on the surface of the molecular model of PP1α were found using an icmPocketFinder algorithm [62]. The cavities grid map was contoured at 4σ level and split into individual cavities. Top 10 biggest cavities were used to determine the docking sites (amino acid residues in 3 Å vicinity). Standard ICM docking was performed for each docking site, as described in [63]. Thoroughness parameter was set to 10. ICM score was calculated for the top 10 positions of the ligand.

### Statistical analysis

Results are expressed as mean ± SD or ±SEM. Differences between any two groups were compared with the unpaired two-tailed Student’s t test on GraphPad Prizm 4.01 software (GraphPad Software, La Jolla, CA, USA).
